# Peptide receptor radionuclide therapy (PRRT) in metastatic neuroendocrine tumors of unknown primary (CUP-NETs)

**DOI:** 10.7150/thno.88619

**Published:** 2024-01-01

**Authors:** Richard P. Baum, Peipei Wang, Vivianne Jakobsson, Tianzhi Zhao, Christiane Schuchardt, Pek-Lan Khong, Jingjing Zhang

**Affiliations:** 1 CURANOSTICUM Wiesbaden-Frankfurt, Center for Advanced Radiomolecular Precision Oncology, Wiesbaden, Germany.; 2 Theranostics Center for Molecular Radiotherapy and Precision Oncology, ENETS Center of Excellence, Zentralklinik Bad Berka, Bad Berka, Germany.; 3 Department of Diagnostic Radiology, Yong Loo Lin School of Medicine, National University of Singapore, Singapore, Singapore.; 4 Clinical Imaging Research Centre, Centre for Translational Medicine, Yong Loo Lin School of Medicine, National University of Singapore, Singapore, Singapore.; 5 Department of Nuclear Medicine, Peking Union Medical College Hospital. Chinese Academy of Medical Science and Peking Union Medical College, Beijing Key Laboratory of Molecular Targeted Diagnosis and Therapy in Nuclear Medicine, Beijing, China.; 6 Nanomedicine Translational Research Program, NUS Center for Nanomedicine, Yong Loo Lin School of Medicine, National University of Singapore, Singapore, Singapore.

**Keywords:** NETs of unknown primary site (CUP-NETs), peptide receptor radionuclide therapy (PRRT), somatostatin receptor (SSTR), efficacy, ^ 177^Lu, ^90^Y

## Abstract

**Rationale:** Peptide receptor radionuclide therapy (PRRT) for the treatment of neuroendocrine tumors (NETs) has been explored for more than two decades, but there are only limited data on the treatment of NETs of unknown primary site (CUP-NETs). This study aimed to analyze the long-term outcome, efficacy, and safety of PRRT in patients with CUP-NETs.

**Methods:** Patients with pathologically confirmed metastatic CUP-NET who received lutetium-177 (^177^Lu) and/or yttrium-90 (^90^Y) labeled somatostatin analogs between March 2001 and March 2019 were retrospectively reviewed; those patients were referred as cCUP-NETs (clinical CUP-NETs). Eighty-one patients had unknown primary tumors even after [^68^Ga]Ga-SSTR and [^18^F]FDG PET/CT and were classified as pCUP-NETs (PET CUP-NETs). Treatment response was assessed according to RECIST 1.1 and PERCIST. Progression-free survival (PFS) and overall survival (OS) were estimated using Kaplan-Meier analysis, and adverse events were graded according to the National Cancer Institute Common Terminology Criteria for Adverse Events (CTCAE), version 5.0.

**Results:** A total of 575 PRRT cycles were administered to 156 patients (76 men and 80 women) evaluable for analysis: these patients were monitored for a median period of 92.3 mo (range, 4.0-169.1 mo). The disease control rate was 41.4% (43.4%) by RECIST and 40.2% (40.8%) by PERCIST in cCUP-NENs (pCUP-NETs). The objective response rate (ORR) with PRRT was 29.4% and 32.2% in cCUP-NENs and pCUP-NETs, respectively. The median PFS and OS for the entire cohort were 17.4 mo (95% confidence interval [95% CI], 11.4-23.4) and 67.4 mo (95% CI, 47.2-87.2) for all patients, respectively. The median OS for G3 tumors was significantly lower (15 mo) than for patients with G1 NET (85.5 mo), G2 (71.7 mo), and for patients with unknown grade (63.3 mo) NETs (P = 0.186, HR: 10.6, 95% CI: 3.87, 28.97, P = 0.09). PRRT was well tolerated by all patients. During treatment and long-term follow-up, CTCAE grade 3 and grade 4 thrombocytopenia and leukocytopenia were observed in only 3 patients (1.9%); there was no evidence of renal or hepatic toxicity.

**Conclusion:** In a large cohort of patients with advanced CUP-NETs treated with PRRT in a real-world scenario and followed up to 14 years after the commencement, PRRT has demonstrated favorable and clinically significant efficacy and survival with minimal and acceptable side effects. Our results indicate that PRRT is a well-tolerated and effective treatment option for patients with metastatic CUP-NETs expressing somatostatin receptors.

## Introduction

Neuroendocrine tumors (NETs) represent a diverse group of malignancies arising from neuroendocrine cells found throughout the body. These tumors exhibit heterogeneous behavior, which contributes to their complex and often unpredictable clinical course 1, 2. The embryogenesis of the diffuse neuroendocrine system accounts for the broad spectrum of these tumors. Gastroenteropancreatic NETs (GEP-NETs) account for approximately two-thirds of all cases, bronchopulmonary NETs (BP-NETs) make up approximately 22-27%, and NETs of unknown primaries (CUP-NETs) occur in 10-20% of cases. Up to 5% arise from endocrine glands, endocrine islets other than the pancreas (thyroid), and in other organs, such as the ovaries and gonads 2-4. The overexpression of somatostatin receptors (SSTRs) in NETs lays the groundwork for the diagnosis (PET/CT) and peptide receptor radionuclide therapy (PRRT) 5.

Recent studies have shown that PRRT could be a highly effective treatment option for patients with metastatic, well-differentiated, or moderately differentiated NETs of known primary origin 6-8. The most robust evidence in favor of PRRT in NETs derives from the phase 3 NETTER-1 trial, which demonstrated in patients with midgut NET, using [^177^Lu]Lu-DOTATATE an objective response rate (ORR) of 18%, compared to 3% in patients receiving high-dose octreotide, with a median progression-free survival (PFS) of 28.4 mo, and overall survival (OS) of 48 mo 9, 10. Beyond the currently most used β-emitting radionuclides ^177^Lu and ^90^Y, labeled to SSTR analogs, promising results have emerged from initial studies using α-emitters and combination therapies 11, 12. These developments could significantly contribute to the more effective treatment of NET patients undergoing PRRT. However, due to the low incidence and tremendous heterogeneity of NETs, despite the promising results of PRRT in patients with known primary tumors 11-15, there is a paucity of data regarding its efficacy and safety in the treatment of metastatic CUP-NETs. This knowledge gap underscores the need for further investigation into the long-term outcomes and potential benefits of PRRT in this specific patient population.

Given the relatively low incidence of CUP-NETs and the heterogeneity of NETs, we intended to systematically collect all data to critically analyze the use of PRRT in those patients. Here, we define two types of CUP-NETs: one group with the clinical diagnosis of tumors of unknown primary before SSTR-targeted imaging (cCUP-NETs), and a second group with still unknown primaries after both, SSTR and [^18^F]FDG PET/CT (pCUP-NETs). Therefore, the primary objective of this study was to evaluate the long-term efficacy and safety of PRRT in those patients.

## Methods

### Study Design and Population

This retrospective study was conducted at Zentralklinik Bad Berka between March 2001 and March 2019 (ENETS Center of Excellence since 2011), analyzing adult patients with metastatic NETs who underwent PRRT. Eligible patients had histopathological confirmation of metastatic NETs with unknown primary tumors at the time of referral for PRRT. All patients included in the study demonstrated abundant SSTR expression as confirmed by SSTR imaging with level 2-3 uptake according to the Krenning scale 15. They received either ^177^Lu or ^90^Y somatostatin analog PRRT under the compassionate use clause of the German Medicinal Products Act. The study was conducted in compliance with German regulations (Federal Agency for Radiation Protection) concerning radiation safety and was approved by the local ethics committee (Bad Berka, Germany). Informed consent was obtained from all patients for the treatment and the use of their anonymized clinical data for scientific purposes. A total of 156 patients with metastatic CUP-NETs were included. These patients are referred to as cCUP-NETs due to the absence of primary tumor detection upon initial conventional diagnosis. Before PRRT, all patients underwent SSTR, and 138 out of 156 patients underwent [^18^F]FDG imaging. Patients who remained with unknown primary tumors after ^111^In SPECT/CT (2001 to 2004), [^68^Ga]Ga- DOTATOC or [^68^Ga]Ga-DOTANOC PET/CT (since 2004), and [^18^F]FDG (since 2003) imaging with both tracers were classified as pCUP-NETs.

### PRRT Regimen

The DOTA-conjugated somatostatin analogs, DOTATOC, DOTANOC, and DOTATATE, HA-TATE, and DOTA-LM3, were radiolabeled with^177^Lu, and ^90^Y, in compliance with good manufacturing practices (GMP) regulations at the institutional radiopharmacy. A radiochemical purity exceeding 98% was consistently achieved. The administration of radiopharmaceuticals adhered to a standardized protocol, as previously described, with more details shown in the supplemental material.

### Safety, Response, and Survival Evaluation

The primary objective of this study was to describe the outcomes (ORR, PFS, and OS) and safety (toxicity) of PRRT in cCUP-NETs and pCUP-NETs. As a secondary objective, other prognostic variables and confounding factors were considered for the effect on OS.

Safety assessment included monitoring laboratory parameters, including routine blood counts (leukocytes, hemoglobin, platelets, and differentials), liver and kidney function parameters, and respective tumor markers, evaluated at baseline, before each treatment cycle, and during restaging. Renal function was monitored using ^99m^Tc-mercaptoacetyltriglycine renography for determining the tubular extraction rate; besides measuring the glomerular filtration rate according to the Cockcroft-Gault Formula. Treatment-related adverse events were graded according to the Common Terminology Criteria for Adverse Events (CTCAE, version 5.0).

The treatment response was assessed on CT and/or MRI according to Response Evaluation Criteria in Solid Tumors (RECIST 1.1) 16 and on molecular imaging ([^68^Ga]Ga-DOTATOC or DOTANOC PET) following the European Organization for Research and Treatment of Cancer (EORTC) criteria (PERCIST 1.1) 17. Imaging was performed before the first PRRT cycle and at restaging every 3 to 6 months after PRRT. In the case of stable disease (SD), partial remission (PR), or complete remission (CR), restaging was performed every 6 mo after the initial follow-up until disease progression (PD) was evident on imaging. Disease control rate (DCR) was defined as the proportion of patients with CR, PR, SD, or mixed remission (MR). The ORR was defined as the proportion of patients achieving CR, PR, or SD during follow-up according to RECIST 1.1. Clinical observations, including lab values, vital signs, symptoms, or self-reported well-being, were also taken into account.

PFS and OS were the primary endpoints of the study. PFS was defined as the time from the initiation of PRRT to the documentation of disease progression or last visit, whereas OS was defined as the time from the initiation of PRRT to death from any cause. Patients without documented progression or death were censored at the time of their last follow-up. After reaching the time point of PFS, OS was evaluated through personal visits to the clinic in the follow-up. All analyses were performed separately for cCUP-NETs and pCUP-NETs patients.

### Statistical analyses

Data were collected by using a prospective database (including over 250 parameters per patient), and categorized based on patient characteristics, tumor characteristics, prior treatments, PRRT radionuclide, PRRT cycle, cumulative activity, and follow-up. The Kaplan-Meier survival analysis was employed to estimate PFS and OS, starting from the initiation of PRRT. To analyze the survival distribution of subgroups, both the log-rank test and the Cox proportional hazards model were utilized. Continuous variables were represented as mean ± standard deviation. Student's t-tests were employed to determine differences between two independent groups. All statistical tests were two-tailed, and a P-value less than 0.05 was considered statistically significant.

## Results

### Patients and treatment

A total of 156 patients with CUP-NENs (76 men and 80 women; age range, 28-85 years; mean age, 60.3 years [SD, 13.1 years]) who underwent PRRT between March 2001 and December 2017 were retrospectively reviewed. **Table 1** provides a comprehensive overview of the patients' baseline clinical characteristics. Primary tumors were detected in 75 patients, with 70 patients showing SSTR positivity and 16 exhibiting FDG positivity. There was no statistical difference between cCUP-NETs and pCUP-NETs in baseline demographic and clinical characteristics. Of these patients, 31 (19.9%) had G1 NETs, 74 (47.4%) had G2 NETs, 9 (5.8%) had G3 NETs and in 42 (26.9%), the proliferation rate (Ki-67 index) remained unknown despite efforts to obtain tissue for pathological analysis.

**Figure 1:**
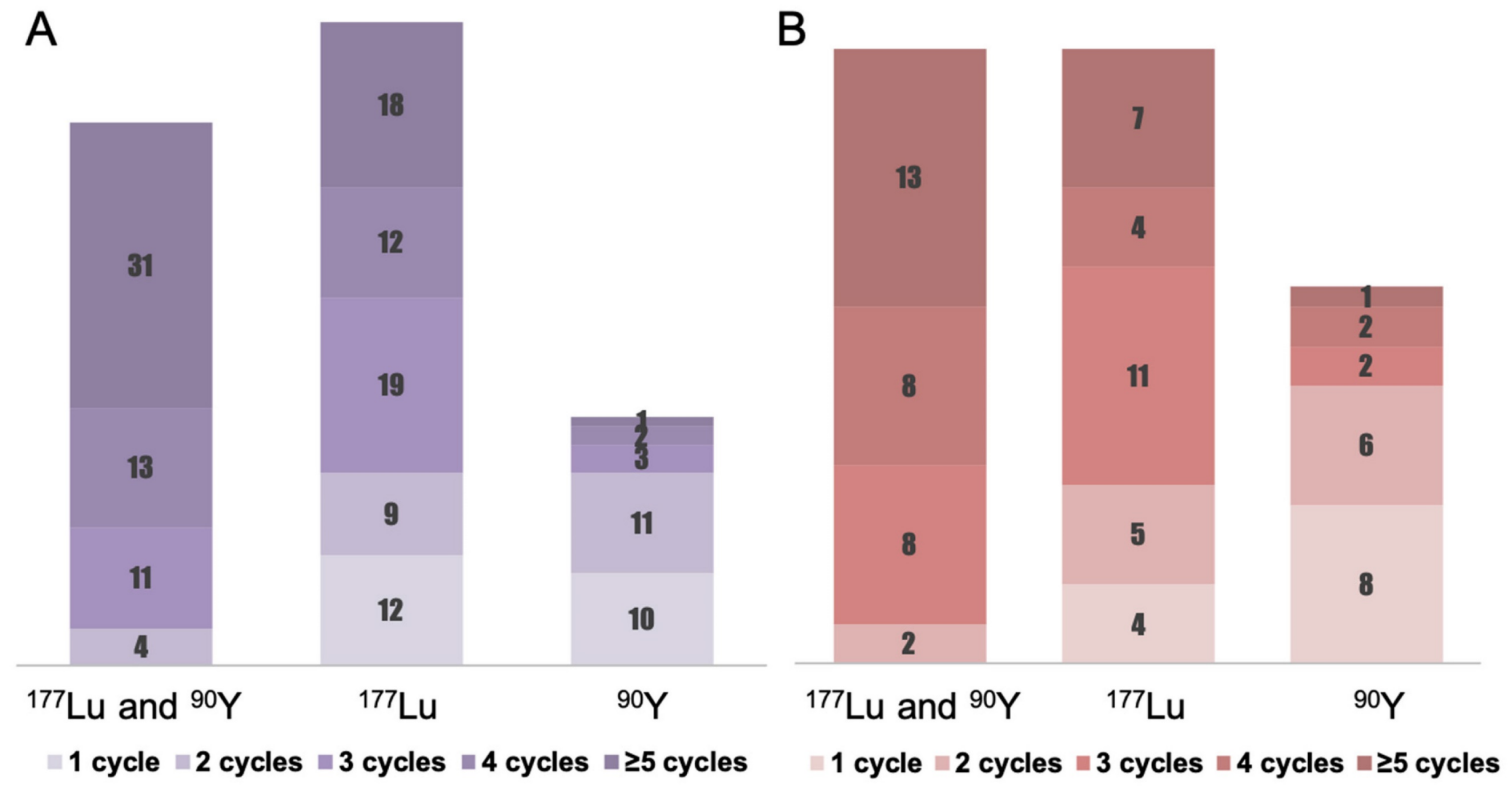
** Detailed PRRT regimen distribution for CUP-NETs.** The columns represent the number of patients who received certain cycles of PRRT. **(A)** Illustrates the PRRT regimen distribution for cCUP-NETs among 156 patients. The regimens include a combination of ^177^Lu and ^90^Y ("TANDEM PRRT") administered to 59 patients (37.8%), ^177^Lu monotherapy to 70 patients (44.9%), and ^90^Y to 27 patients (17.3%). **(B)** Portrays the PRRT regimen distribution for pCUP-NETs among 81 patients, which comprises the "TANDEM PRRT" combination to 31 patients (38.3%), ^177^Lu monotherapy to 31 patients (38.3%), and ^90^Y to 19 patients (23.4%).

**Figure 1, Table 2, and Supplemental Table 1** present the treatment parameters and PRRT cycle distribution. A total of 575 PRRT courses were given to the 156 patients. 49.4% of patients received four or more cycles of PRRT, with 1.9% (n = 3) treated with **^90^**Y, 19.2% (n = 30) with ^177^Lu, and 28.2% (n = 44) with a combination of ^177^Lu and ^90^Y (“TANDEM PRRT”). The mean cumulative administered radioactivity for all patients was 21.2 ± 13.8 GBq (range 2.5 - 78.7 GBq); for pCUP-NETs, the mean cumulative administered radioactivity was 19.1 ± 13.3 GBq (range 2.5 - 78.6 GBq).

### Safety

Patients in this study tolerated PRRT remarkably well, with no severe clinically significant acute or long-term toxicities observed (**Table 3**). Grade 3 or 4 hematotoxicities were observed 6-18 mo after at least three administrations in three (1.9%) patients. One patient developed grade 4 leukocytopenia and grade 3 thrombocytopenia one year after receiving 6 cycles of PRRT (4.5 GBq ^90^Y and 34.8 GBq ^177^Lu). Another patient experienced grade 4 thrombocytopenia 6 mo after 3 cycles of PRRT (16.7 GBq ^177^Lu). One patient presented with grade 3 leukopenia 1.5 years after completing 4 cycles of PRRT (25.2 GBq ^177^Lu). At baseline, 9 patients exhibited grade 2 anemia, which did not worsen following PRRT. After treatment, 19 patients developed grade 2 anemia, with one patient having normal hemoglobin values and 14 patients having grade 1 anemia at baseline. Four patients had grade 2 anemia at baseline. The post-treatment anemia was possibly related to disease progression in the bone marrow due to disseminated bone metastases. No grade 3 or 4 anemia was observed after PRRT, although 1 patient (0.6%) had grade 3 anemia before PRRT.

No CTCAE grade 3 or 4 nephrotoxicity or hepatotoxicity was observed in any patient. At baseline, creatinine levels were normal in 126 of 156 patients (80.8%), whereas 16.7% (26/156) had grade 1 and 2.6% (4/156) had grade 2 renal dysfunction. On follow-up, 67.9% (106/155) had normal creatinine, 28.2% (44/155) had grade 1, and 3.2% (5/155) had grade 2 elevation of creatinine. However, there was no correlation between the number of cycles or the cumulative administered radioactivity. Notably, no patients with grade 2 renal impairment at baseline demonstrated a further decline in renal function.

### Response

**Figure 2:**
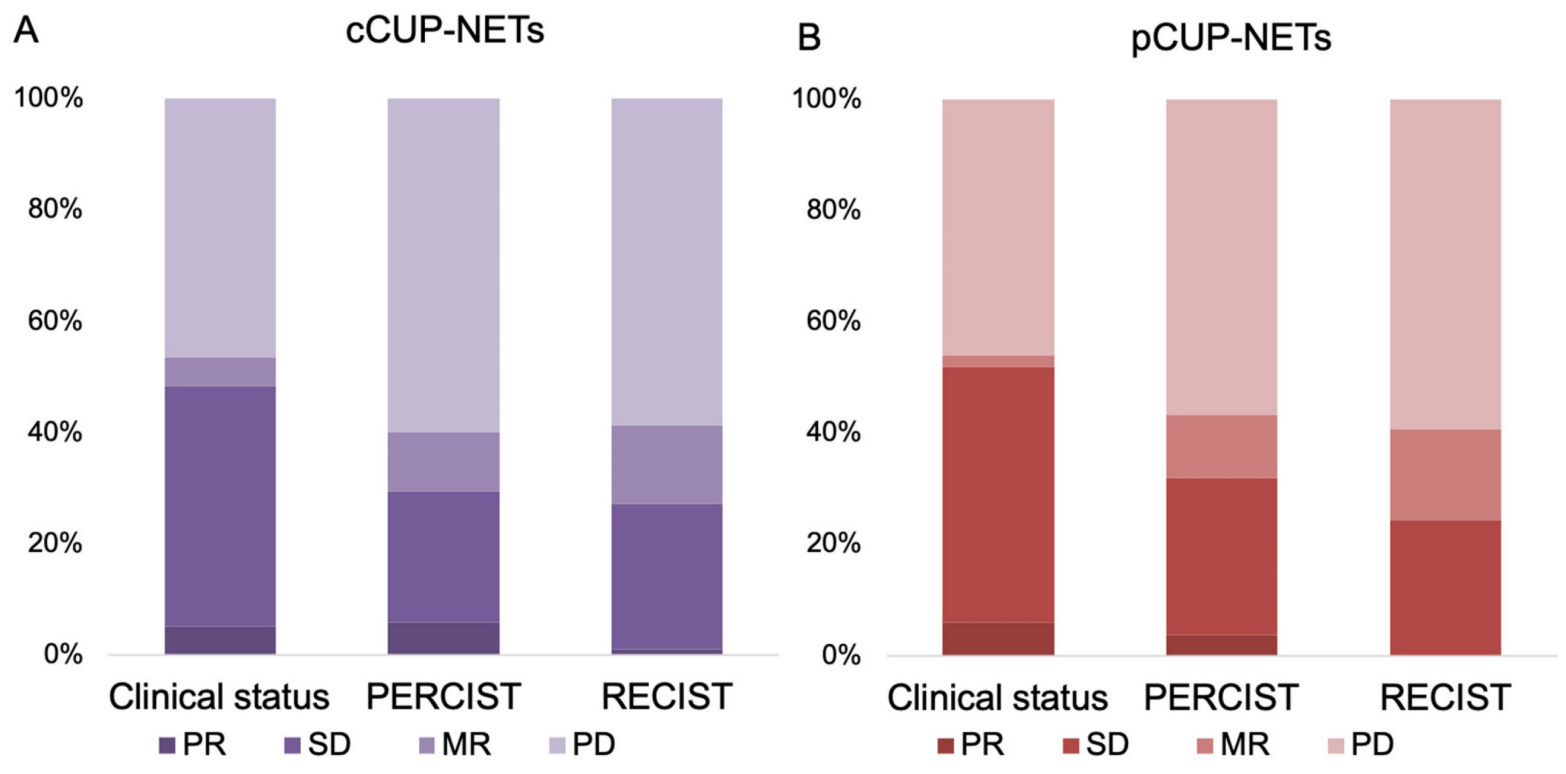
**Response biodistribution of CUP-NETs, assessed with clinical interview, SSR imaging, and with anatomical imaging. (A)** cCUP-NETs: Data available for 62-65% (n = 97-102) of 156 cases. The best RECIST1.1 response recorded a PR in 1% (n = 1) of cases, and PERCIST observed a PR in 5.9% (n = 6) of cases. DCR were 43.4% (PERCIST) and 40.8% (RECIST). **(B)** pCUP-NETs: Data available for 62-65% (n = 97-102) of cases. The best response in RECIST1.1 observed SD in 24.5% (n = 12) of cases, with PERCIST noting a PR in 3.8% (n = 2) of cases.

In the current study, the response evaluation for CUP-NETs patients was based on clinical observations, PERCIST, and RECIST criteria. Among these patients, response data were available for 62% (n = 97), 65% (n = 102), and 63% (n = 99) of the cases, respectively, as shown in **Figure 2 and Supplemental Table 2.** In the subset of patients with measurable and response-evaluable cCUP-NETs (99/156), the best RECIST1.1 response was PR in 1.0% (n = 1), SD in 26.3% (n = 26), MR in 14.1% (n = 14), and PD in 58.6% (n = 58), respectively. The DCR was 41.4% according to RECIST, and 40.2% according to PERCIST. The latter comprised 5.9% (n = 6) PR, 23.5% (n = 24) SD, and 10.7% (n = 11) MR, respectively. Consequently, the ORR was 29.4% based on PERCIST and 27.3% based on RECIST criteria.

For pCUP-NETs patients, response evaluation was similarly based on clinical observations, PERCIST, and RECIST criteria, with response data available for 61% (n = 50), 65% (n = 53), and 60% (n = 49) of the cases, respectively. The DCR was 43.4% according to PERCIST, and 40.8% according to RECIST. The ORR was 32.1% based on PERCIST and 24.5% based on RECIST criteria.

### Survival

**Figure 3:**
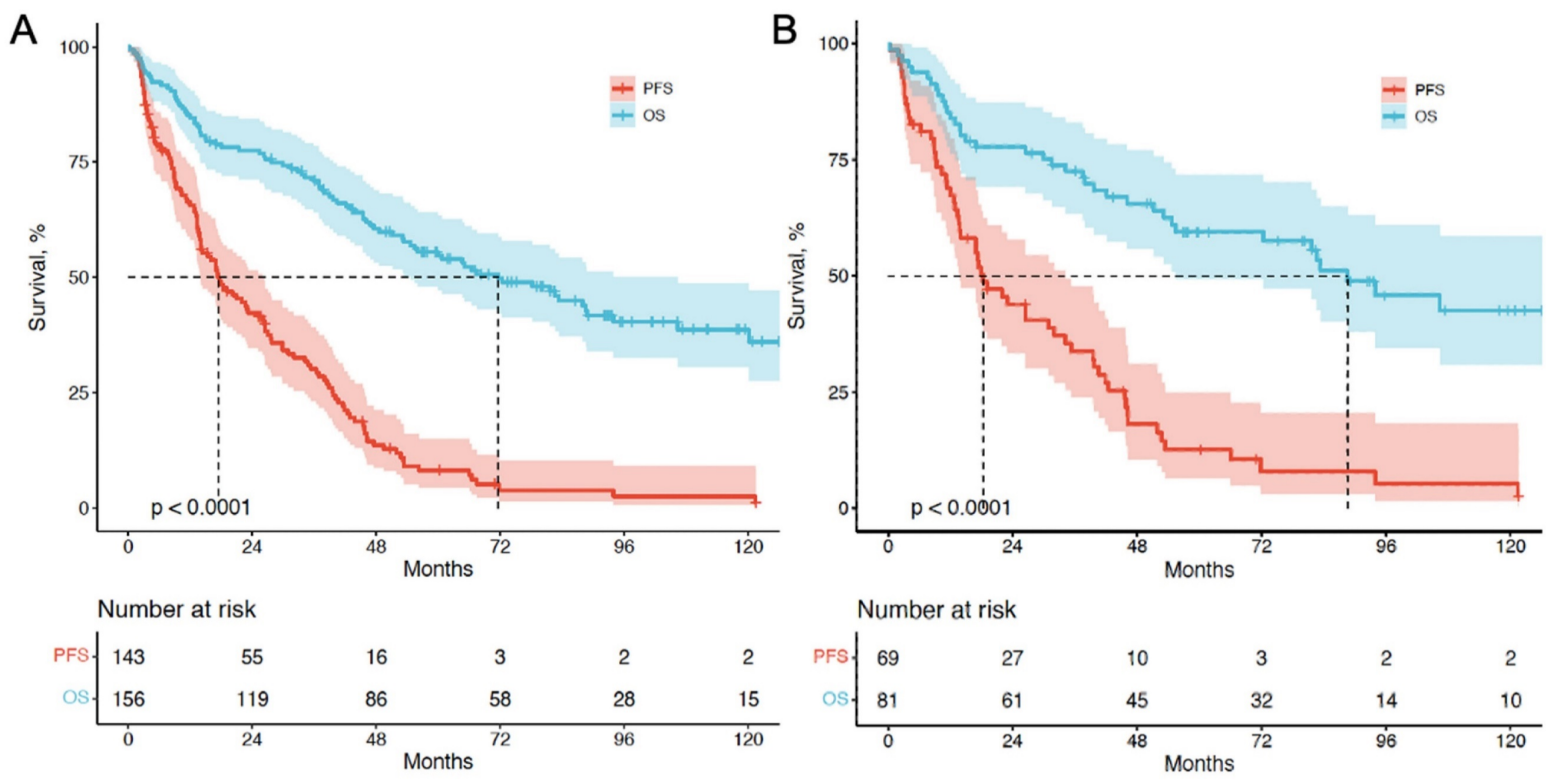
** Kaplan-Meier curves depicting PFS and OS (in months) for patients with CUP-NETs from PRRT initiation. (A)** CUP-NETs patients' survival outcomes, with a median follow-up period of 92.3 months (4.0-169.1 months), a median PFS of 17.4 months (11.4-23.4 months), and a median OS of 71.6 months (50.3-92.9 months). **(B)** pCUP-NETs patients' survival outcomes, revealing a median PFS of 18.3 months (11.7-24.8 months) and a median OS of 88.6 months (64.8-112.3 months).

For survival analysis, data were assessed until the study cutoff date in March 2019. The median follow-up time was 93.4 mo (range, 4.0-169.1mo). Among the 156 patients with cCUP-NETs, 85 (54.5%) died, while 38 out of 81 patients (46.9%) with pCUP-NETs died. Five- and ten-year OS were 55.6% and 45.5%, respectively. For the entire group of 156 patients, the median PFS was 17.4 mo (95% CI, 11.4-23.4), and the median OS was 67.4 mo (95% CI, 47.2-87.2). Among the 81 patients with pCUP-NETs, the median PFS was 18.3 mo (95% CI, 11.7-24.8), and the median OS was 88.6 mo (95% CI, 64.8-112.3) (**Figure 3**).

**Figure 4:**
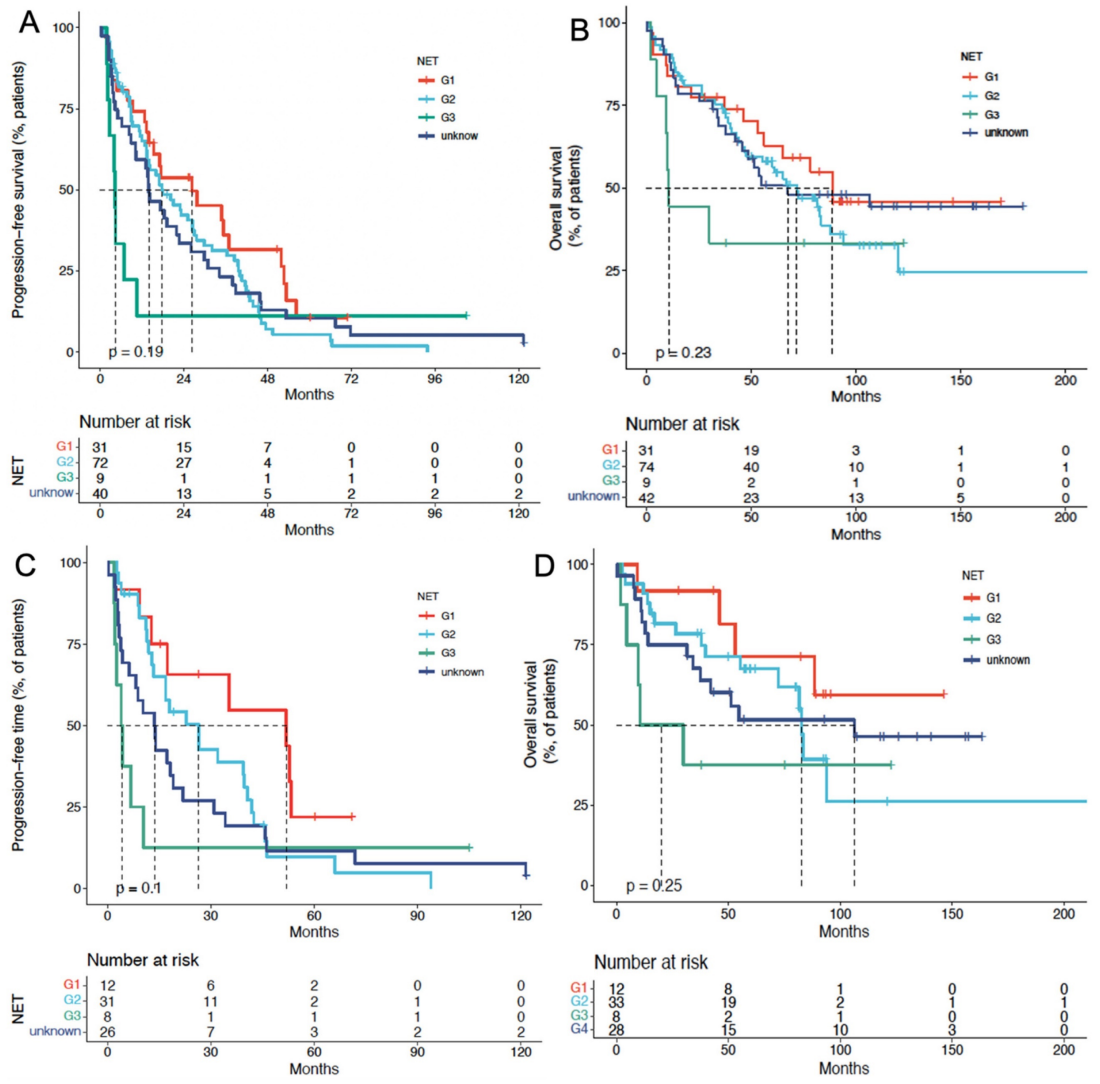
**PFS and OS of PRRT in patients with CUP-NETs, differentiated by tumor grade (G1, G2, G3, and unknown). (A)** Median PFS in cCUP-NETs: 25.0 months (G1), 19.2 months (G2), 6.4 months (G3), and 15.6 months (unknown) (P = 0.335). **(B)** Median OS in cCUP-NETs: 85.5 months (G1), 71.7 months (G2), 15 months (G3), and 63.3 months (unknown) (P = 0.186). (C) Median PFS in pCUP-NETs: 51.5 months (G1), 23.1 months (G2), 6.6 months (G3), and 12.9 months (unknown) (P = 0.04). (D) Median OS in pCUP-NETs: 140 months (G1), 85.5 months (G2), 20.0 months (G3), and 102.9 months (unknown) (P = 0.119).

In the cCUP-NETs group, the median PFS for G1, G2, G3, and unknown NETs were 25.0 mo, 19.2 mo, 6.4 mo, and 15.6 mo (P = 0.335), respectively. The median OS for G1, G2, G3, and unknown NETs were 85.5 mo, 71.7 mo, 15 mo, and 63.3 mo (P = 0.186), respectively, with a P-value of 0.093 for the comparison between G1, G2, and G3 subgroups. (**Figures 4A and 4B**) Cox analysis showed that G3 patients had a higher risk concerning OS, with an HR of 10.6 (95% CI, 3.87-28.97).

In the pCUP-NETs group, the median PFS for G1, G2, G3, and unknown Ki-67 index CUP-NETs were 51.5 mo, 23.1 mo, 6.6 mo, and 12.9 mo (P = 0.04), respectively. The median OS for these subgroups was 140 mo, 85.5 mo, 20.0 mo, and 102.9 mo (P = 0.119), respectively. Comparing G1, G2, and G3 subgroups yielded a P-value of 0.046 (**Figures 4C and 4D**).

## Discussion

In this retrospective study, we investigated the safety, treatment response, and survival outcomes of PRRT in patients with cCUP-NENs and pCUP-NETs. The results of this analysis in a well-characterized cohort of 156 patients show that PRRT was well-tolerated, with minimal severe acute or long-term toxicity. Moreover, during the median follow-up time of 93.4 mo (range, 4.0-169.1 mo), we observed disease control rates of 41.4% in cCUP-NETs, 43.4% in pCUP-NETs according to RECIST, along with favorable survival outcomes, with a median PFS of 17.4 mo, and a median OS of 67.4 mo in the entire CUP-NET patient cohort. According to the current WHO grading with a Ki-67 cut-off value of 2% and 20% (G1 *vs.* G2 *vs.* G3), higher grading was associated with a shorter PFS and OS in our cohort (PFS, 25.0 *vs.* 19.2 *vs.* 6.4; OS, 85.5 *vs.* 71.7 *vs.* 15; HR for G3 OS, 10.6). These results confirm the potential of PRRT as a promising treatment option for patients with CUP-NENs.

Most PRRT studies have focused on well-differentiated NETs with known primary sites. Consideration of the site of the primary tumor is considered important in determining if a patient should be treated with PRRT 14, 18, 19. Recent research has discussed the use of PRRT for the treatment of NET subtypes, assuming that the disease is SSTR-positive on SSTR PET/CT. Mitjavila *et al.* enrolled 522 subjects with pancreatic (35%), midgut (28%), bronchopulmonary (11%), pheochromocytoma/paraganglioma (6%), other GEP-NET (11%), and non-GEP (9%) NENs and found a PFS of 19.8 mo, 31.3 mo, 17.6 mo, 30.6 mo, 24.3 mo, and 20.5 mo, respectively 8. Regardless of anatomical location, a wide range of SSTR-expressing NETs benefit from PRRT with similar survival outcomes, and tumors of unknown primary are less common since the introduction of SSTR-PET. In a study conducted by Brabander *et al*, 82 patients with NETs of unknown primary were included; the ORR was 35%, the median PFS of 29 mo, and an OS of 53 mo was found 20. However, detailed patient characteristics and treatment details were not clearly described. Other studies related to CUP-NETs have only included a few cases 21, 22. To date, there are no prospective PRRT studies published in patients with tumors of unknown primaries only, as affirmed by a recent critical review by Urso *et al.*, which summarized the literature on the use of Lutathera outside approved indications, including the above-mentioned ones for CUP-NET patients 23.

The demographic and clinical characteristics of patients with pCUP-NETs and cCUP-NETs were similar, which may suggest a similarity between CUP-NETs and a wide range of SSTR-positive NETs in terms of PRRT selection and therapeutic efficacy. In a systematic review of 500 patients with CUP-NETs 24, the results also showed that well-differentiated CUP-NETs are associated with a more indolent course and a survival range resembling that of typical and atypical pulmonary, and well-differentiated GEP-NETs. To the best of our knowledge, the present study includes the largest cohort of patients with CUP-NETs treated with PRRT so far. All patients exhibited varying degrees of metastases, with 65 patients (41.6%) not having undergone any systemic treatment before PRRT and 41 patients (26.3%) choosing PRRT as their first-line treatment strategy. No evidence for distinct biology or outcome of CUP-NETs patients emerged when the histological grade was matched for known primary NETs.

In the NETTER-1 phase 3 trial, grade 3 or 4 neutropenia, thrombocytopenia, and lymphopenia occurred in 1%, 2%, and 9% of the cases, respectively 9. In this study, safety outcomes revealed that patients generally tolerated PRRT well, with only 3 cases (1.9%) exhibiting grade 3 or 4 hematotoxicity, 6-18 mo after at least 3 cycles. Grade 3 or 4 thrombocytopenia and lymphopenia both occurred in 1.2% of the cases. These findings are consistent with previous studies that have reported limited acute and long-term toxicities associated with PRRT. Furthermore, we observed no significant nephrotoxicity or hepatotoxicity in our patient cohort, which is also in line with previously reported PRRT studies 6, 25, 26. Overall, our results suggest that PRRT is a safe treatment option for patients with CUP-NETs.

The response rates observed in our study were comparable to those of patients with midgut NETs and bronchial neuroendocrine tumors but lower than those of patients with pancreatic NET as reported in the literature 13. We found that the DCR was 41.4%, with a 27.3% ORR for cCUP-NETs according to RECIST. Brabander *et al.* demonstrated the highest ORR of 55% in patients with pancreatic NETs (in 133 patients), with a median PFS and OS of 30 and 71 mo, respectively, which were higher than those with NETs of other primaries 20. However, the SEPTRALU trial 8, focusing on NETs with different primary sites, showed a median PFS of 19.8 mo for pancreatic NETs, which was lower than that for GEP-NETs (31.3 mo), with comparable ORR. This discrepancy could be attributed to variations in tumor burden and the heterogeneity of tumor and patient clinical characteristics across different centers. We observed that Brabander *et al.* reported a higher DCR in a study involving 82 patients (78%), as Demirci *et al.* did in their research on 19 patients (84.2%). Both studies were conducted on mixed cohorts of SSTR-positive CUP-NETs treated with PRRT 20, 27. Yet, the detailed characteristics of CUP-NETs patients and the specifics of their treatment were not delineated sufficiently to allow a direct comparison to our findings. The lack of an identified primary site might serve as a negative prognostic indicator due to the subsequent restriction in therapeutic choices 28. Notably, in our investigation, SSTR-targeted imaging identified the concealed primary tumor in just 48.1% (75/156) of CUP-NET patients, which is consistent with a previous study with a 59% detection rate for CUP-NET 29. This limitation suggests that certain lesions remain undetectable and thus untreatable by SSTR. This might account for the diminished response relative to patients with a discernible primary tumor.

Our study indicates that PRRT also exhibits considerable efficacy in treating CUP-NETs, potentially mirroring the outcomes seen in other types of NETs. Furthermore, our findings revealed no significant differences in ORR (27.3% *vs.* 24.5%) and PFS (17.4 mo *vs.* 18.3 mo) between the cCUP-NETs and pCUP-NETs groups, which suggests that, in terms of tumor characteristics, clinical management, and treatment, CUP-NETs align with NETs of known primary origin. However, the PFS of CUP-NETs is lower than that of other types of NETs, of which the median PFS ranges from 20 to 59 mo 30, 31. The median OS in our study is comparable to previous studies, ranging from 34 to 84 mo 32. However, significant differences were found in survival among patients with different WHO gradings of NETs, where G3 CUP-NETs had the lowest PFS and OS of 15 mo. A previous study has shown a similar trend that patients who had a Ki-67 index of less than 35% had a significantly longer PFS than that of patients with a Ki-67 index of greater than 35% (6.8 and 26.3 mo, P = 0.005) 33; Ki-67 is generally a good indication of the prognosis of NETs. However, compared with other treatment modalities, PRRT has demonstrated promising survival outcomes even in G3 NET patients 34. Another study supporting these findings was conducted by Zhang *et al.*, who reported a large cohort of G3 NET patients and found a median PFS of 9.6 mo and a median OS of 19.9 mo 26.

There are several limitations to our study. First, the retrospective nature of the study may have introduced a selection bias (despite prospective data sampling in a systematic NET database with over 250 parameters per patient) as patients who were referred for PRRT might have had more favorable baseline characteristics compared to those who were not. Second, our study cohort included patients with heterogeneous disease characteristics, which may have contributed to the variability in treatment response and survival outcomes observed in our analysis. Lastly, it is essential to acknowledge the heterogeneity of radiopharmaceuticals used in our patient cohort due to the retrospective nature of the study and the real-world scenario. The variations in the types of radiopharmaceuticals used for both diagnostic imaging and PRRT treatment were inevitable, considering the timeframe of the study and the evolving landscape of nuclear medicine. However, it is important to highlight that despite these variations, the imaging modalities with ^111^In SPECT/CT (2001 to 2004) or [^68^Ga]Ga-DOTATOC or DOTANOC-PET/CT (since 2004) have demonstrated effectiveness in selecting patients for PRRT. Additionally, PRRT using different compounds of DOTATOC and DOTATATE, which comprised the vast majority of treatments in this study (97.9%), radiolabeled with both ^90^Y and ^177^Lu, have been widely used in clinical research and proven to be effective and safe for NET. Given the real-world nature of our study and the inclusion of a large cohort of patients with known primary tumors, our findings provide valuable and solid data to investigate the long-term outcomes of PRRT in patients with CUP-NET.

Despite these limitations, our study provides valuable insights into the safety, response, and survival outcomes of PRRT in patients with CUP-NENs, showing that PRRT is a well-tolerated and effective treatment option for these patients, with favorable disease control rates and overall survival outcomes. Further research and prospective clinical trials are needed to identify the most effective radioisotopes and peptides (*e.g.*, antagonists) for PRRT of patients with CUP-NETs.

## Supplementary Material

Supplementary methods and tables.

## Figures and Tables

**Table 1:** Demographic and Baseline Clinical Characteristics of Patients With CUP-NETs

Baseline characteristic	cCUP-NETs, N = 156 (100%)	pCUP-NETs, N = 81 (100%)
Gender — no. (%)		
Men	76 (48.7)	42 (51.9)
Women	80 (51.3)	39 (48.1)
Age (years), median (range)	60.3 (27.6 - 85.2)	61.2 (32.3 - 85.2)
≤ 45 y	16 (10.3)	8 (9.9)
> 45 y	140 (89.7)	73 (90.1)
Ki-67%, median (range)	9.3±12.8 (1-70)	12.7±15.8 (1-70)
Missing	58	29
WHO 2017 — no. (%)		
NET G1	31 (19.9)	12 (14.8)
NET G2	74 (47.4)	33 (40.7)
NET G3	9 (5.8)	8 (9.9)
unknown	42 (26.9)	28 (34.6)
Localization of metastases		
Liver	129 (82.7)	66 (81.5)
Lymph nodes	91 (58.3)	41 (50.6)
Bone	63 (40.4)	31 (38.3)
Lung	15 (9.6)	6 (7.4)
Peritoneum	12 (7.7)	8 (9.9)
Other	39 (25.0)	24 (29.6)
Number of Metastases sites		
1	39 (25.0)	24 (29.6)
2	65 (41.7)	35 (43.2)
>2	52 (33.3)	22 (28.2)
Pretreatment		
Surgery	53 (34.0)	20 (24.7%)
Somatostatin analogues	66 (42.3)	32 (39.5%)
Chemotherapy	16 (10.3)	6 (7.4%)
Radiotherapy	13 (8.3)	7 (8.6%)
Other medicine	21 (13.5)	9 (11.1%)
Ablation	16 (10.3)	6 (7.4%)
PRRT	8 (5.1)	4 (4.9%)
Number of prior systemic treatments		
0	65 (41.6)	35 (43.2)
1	66 (42.3)	30 (37.0)
2	23 (14.7)	15 (18.5)
>2	12 (7.7)	8 (9.9)
Number of previous lines		
0	41 (26.3)	21 (25.9)
1	53 (34.0)	31 (38.3)
2	33 (21.1)	15 (9.9)
>2	29 (18.6)	14 (9.9)
Median time from initial diagnosis to PRRT, mo (range)	27.4 ± 37.2 (1.3~193.9, IQR 4.6, 31.9)	29.0 ± 39.4 (1.3~162.9, IQR 4.6, 42.3)
SRI, Krenning scale		
FDG and SRI		
Mismatch	94 (60.3)	44 (54.3)
Match	20 (12.8)	14 (17.3)
Reverse mismatch	24 (15.4)	6 (7.4)
Unknown	18 (11.5)	17 (21.0)
Functional		
Yes	61 (39.1)	34 (42.0)
No	95 (60.9)	47 (58.0)
Symptoms at disease		
Yes	85 (54.5)	43 (53.1)
No	60 (38.5)	33 (40.7)
Unknown	11 (7.0)	5 (6.2)

**Table 2:** Details on PRRT of different SSTR-targeted radiopharmaceutical, cycles, patients, time frame, and administrated activity

Radiopharmaceuticals	Cycles (no.)	Patients (no.)	Time frame (yr)	Activity (GBq)
^90^Y-DOTA-TOC	50	36	2001 - 2018	3.6 ±1.0
^90^Y-DOTA-TATE	111	62	2003 - 2012	3.2 ± 1.1
^90^Y-DOTA-NOC	3	3	2004 - 2004	3.3 ± 0.6
^177^Lu-DOTA-TOC	194	82	2009 - 2018	6.6 ± 1.0
^177^Lu-DOTA-TATE	142	71	2004 - 2014	6.4 ± 1.3
^177^Lu-DOTA-NOC	3	3	2009 - 2004	6.1 ± 1.6
^177^Lu-HA-TATE	66	32	2013 - 2015	6.5 ± 1.0
^177^Lu-DOTA-LM3	6	3	2017 - 2019	5.8 ± 0.9

**Table 3:** Number of Patients with Adverse Events According to the Common Terminology Criteria for Adverse Events

Baseline	G0	G1	G2	G3	G4	NA
Leukocytes	122 (78.2)	16 (10.3)	2 (1.3)	0	0	0
Thrombocytes	134 (85.9)	6 (3.8)	1 (0.6)	0	0	0
Hemoglobin	48 (30.8)	98 (62.8)	9 (5.8)	1(0.6)	0	0
GOT	121 (77.6)	33 (21.2)	2 (1.3)	0	0	0
GPT	126 (80.8)	26 (16.7)	4 (2.6)	0	0	0
Albumin	119 (76.3)	21 (13.5)	0	0	0	16 (10.3)
Bilirubin	145 (92.9)	5 (3.2)	0	0	0	6 (3.8)
Creatinine	126 (80.8)	26 (16.7)	4 (2.6)	0	0	
After PRRT						
Leukocytes	116 (74.4)	23 (14.7)	6 (3.8)	1 (0.6)	1 (0.6)	1(0.6)
Thrombocytes	110 (70.5)	36 (23.1)	3 (1.9)	1 (0.6)	1 (0.6)	1 (0.6)
Hemoglobin	19 (12.2)	117 (75.0)	19 (12.2)	0	0	1 (0.6)
GOT	110 (70.5)	42 (26.9)	3 (1.9)	1 (0.6)	0	0
GPT	130 (83.3)	22 (14.1)	3 (1.9)	1 (0.6)	0	0
Albumin	114 (73.1)	31 (19.9)	0	0	0	11 (7.1)
Bilirubin	142 (91.0)	7 (4.5)	0	0	0	7 (4.5)
Creatinine	106 (67.9)	44 (28.2)	5 (3.2)	0	0	1(0.6)

## Abbreviations

PRRT: Peptide Receptor Radionuclide Therapy
CUP-NETs: Neuroendocrine Tumors of Unknown Primary
^177^Lu: lutetium-177
^90^Y: yttrium-90
^111^In: indium-111
^68^Ga: gallium-68
DOTATOC: 1,4,7,10-tetraazacyclododecane-1,4,7,10-tetraacetic acid - [Tyr3]-octreotide
DOTATOC: 1,4,7,10-tetraazacyclododecane-1,4,7,10-tetraacetic acid - [Nal3]-octreotide
DOTATATE: 1,4,7,10-tetraazacyclododecane-1,4,7,10-tetraacetic acid - [Tyr3, Thr8]-octreotate
HA-TATE: hydroxyapatite - [Tyr3]-octreotate
DOTA-LM3: 1,4,7,10-tetraazacyclododecane-1,4,7,10-tetraacetic acid - [Lys40(Ahx-NODAGA)NH2]-DOTA-Tyr3-octreotate
GMP: good manufacturing practices
SSTR: somatostatin receptors
FDG: fluorodeoxyglucose
PET/CT: Positron Emission Tomography/Computed Tomography
RECIST: Response Evaluation Criteria in Solid Tumors
PERCIST: Positron Emission Tomography Response Criteria in Solid Tumors
CTCAE: Common Terminology Criteria for Adverse Events
DCR: disease control rate
ORR: objective response rate
PFS: progression free survival
OS: overall survival
CR: complete response
PR: partial response
SD: stable disease
PD: progressive disease
MR: mixed remission
