# Supplementary material for: Peptide receptor radionuclide therapy (PRRT) in metastatic neuroendocrine tumors of unknown primary (CUP-NETs)
Source: Theranostics. 2024 Jan 1;14(1):133–42. doi: 10.7150/thno.88619 (PMC10750196; doi:10.7150/thno.88619)
Supplement: Supplementary file 1 — Supplementary methods and tables. [file thnov14p0133s1.pdf]

Peptide Receptor Radionuclide Therapy (PRRT) in Metastatic Neuroendocrine Tumors of Unknown Primary (CUP-NETs)

Supplemental Material

METHODS

PRRT Regimen

The DOTA-conjugated somatostatin analogs, DOTATOC, DOTANOC, and DOTATATE, HA-TATE, and DOTA-LM3, were radiolabeled with^{177}Lu, and ^{90}Y, in compliance with good manufacturing practices (GMP) regulations at the institutional radiopharmacy. A radiochemical purity exceeding 98% was consistently achieved. The administration of radiopharmaceuticals adhered to a standardized protocol, as previously described. Renal protection was ensured through a custom-prepared lysine and arginine cocktail (1600 mL of 5% lysine·HCl and 10% L-arginine·HCl), administered 30 minutes before and continued for 4 hours after radiopharmaceutical infusion. Additional kidney protection for patients with impaired renal function (glomerular filtration rate <60 mL/min) or those treated with ^{90}Y involved a bolus of 4% Gelofusine (B. Braun Melsungen AG) followed by a continuous infusion. The radiopharmaceutical was co-administered over a 10- to 15-minute period utilizing a secondary infusion pump system. The selection of ^{90}Y or ^{177}Lu, as well as the administered activity, was determined on an individual basis according to the Bad Berka Score. Treatment cycles were spaced at 10 to 12 weeks intervals to allow for optimal patient response and recovery.

Supplemental Table 1 Cycles and Cumulative Administered Radioactivity for PRRT with ^{177}Lu, ^{90}Y, and Both Radionuclides

	cCUP-NETs (n=156)	pCUP-NETs (n=81)
PRRT, n (%)		
^{90}Y (1~6 cycles)	27 (17.3)	19 (23.4)
^{177}Lu (1~11 cycles)	70 (44.9)	31 (38.3)
^{177}Lu and ^{90}Y (2~10 cycles)	59 (37.8)	31 (38.3)
Cumulative Activity, GBq		
^{90}Y	7.2 ± 3.8 (2.5-16.4)	7.0 ± 3.8 (2.5 - 15.3)
^{177}Lu	22.1 ± 13.0 (3.0-78.7)	21.2 ± 10.6 (5.0 - 44.5)
^{177}Lu and ^{90}Y	26.5 ± 13.6 (8.5-78.6)	13.3 ± 3.1 (8.5 - 78.6)
Cycles, n (%)		
1	22 (14.1)	12 (14.8)
2	24 (15.4)	13 (16.0)
3	33 (21.2)	21 (25.9)
4	27 (17.3)	14 (17.3)
>4	50 (32.0)	21 (25.9)

Supplemental Table 2 Treatment Response at 3 Mo After PRRT

Response	cCUP-NETs, N = 99 n (%)	pCUP-NETs, N = 81 n (%)
SSTR imaging response		
CR		0
PR	6 (5.9)	2 (3.8)
SD	24 (23.5)	15 (28.3)
MR	11 (10.7)	6 (11.3)
PD	61 (59.8)	30 (56.6)
DCR	41 (40.2)	23 (43.4)
CT or MRI response		
CR	0	0
PR	1(1.0)	0
SD	26 (26.3)	12 (24.5)
MR	14 (14.1)	8 (16.3)
PD	58 (58.6)	29 (59.2)
DCR	41 (41.4)	20 (40.8)
